# Assessment of Smoke Contamination in Grapevine Berries and Taint in Wines Due to Bushfires Using a Low-Cost E-Nose and an Artificial Intelligence Approach

**DOI:** 10.3390/s20185108

**Published:** 2020-09-08

**Authors:** Sigfredo Fuentes, Vasiliki Summerson, Claudia Gonzalez Viejo, Eden Tongson, Nir Lipovetzky, Kerry L. Wilkinson, Colleen Szeto, Ranjith R. Unnithan

**Affiliations:** 1 Digital Agriculture, Food and Wine Sciences Group, School of Agriculture and Food, Faculty of Veterinary and Agricultural Sciences, The University of Melbourne, Parkville, VIC 3010, Australia; sfuentes@unimelb.edu.au (S.F.); vsummerson@student.unimelb.edu.au (V.S.); eden.tongson@unimelb.edu.au (E.T.); 2 School of Computing and Information Systems, Melbourne School of Engineering, The University of Melbourne, Parkville, VIC 3010, Australia; nir.lipovetzky@unimelb.edu.au; 3 School of Agriculture, Food and Wine, The University of Adelaide, Waite Campus, PMB 1, Glen Osmond, SA 5064, Australia; kerry.wilkinson@adelaide.edu.au (K.L.W.); colleen.szeto@adelaide.edu.au (C.S.); 4 The Australian Research Council Training Centre for Innovative Wine Production, PMB 1, Glen Osmond, SA 5064, Australia; 5 School of Engineering, Department of Electrical and Electronic Engineering, The University of Melbourne, Parkville, VIC 3010, Australia; r.ranjith@unimelb.edu.au

**Keywords:** climate change, machine learning, electronic nose, smoke taint, wine sensory

## Abstract

Bushfires are increasing in number and intensity due to climate change. A newly developed low-cost electronic nose (e-nose) was tested on wines made from grapevines exposed to smoke in field trials. E-nose readings were obtained from wines from five experimental treatments: (i) low-density smoke exposure (LS), (ii) high-density smoke exposure (HS), (iii) high-density smoke exposure with in-canopy misting (HSM), and two controls: (iv) control (C; no smoke treatment) and (v) control with in-canopy misting (CM; no smoke treatment). These e-nose readings were used as inputs for machine learning algorithms to obtain a classification model, with treatments as targets and seven neurons, with 97% accuracy in the classification of 300 samples into treatments as targets (Model 1). Models 2 to 4 used 10 neurons, with 20 glycoconjugates and 10 volatile phenols as targets, measured: in berries one hour after smoke (Model 2; R = 0.98; R^2^ = 0.95; b = 0.97); in berries at harvest (Model 3; R = 0.99; R^2^ = 0.97; b = 0.96); in wines (Model 4; R = 0.99; R^2^ = 0.98; b = 0.98). Model 5 was based on the intensity of 12 wine descriptors determined via a consumer sensory test (Model 5; R = 0.98; R^2^ = 0.96; b = 0.97). These models could be used by winemakers to assess near real-time smoke contamination levels and to implement amelioration strategies to minimize smoke taint in wines following bushfires.

## 1. Introduction

When bushfires occur within the grape growing season, vineyards can be affected at critical stages (véraison to harvest) [1], which could result in different levels of smoke contamination in berries and smoke taint in wines [2,3]. The intensity, number, and severity of bushfires are increasing due to climate change as well as the window of opportunity [4].

The growing concerns in Australia regarding bushfire scale and frequency are shared by wine regions around the world, including the USA, Canada, South Africa, Portugal, Chile, and others [5]. To assess the potential risk of smoke taint, the industry typically relies on the analysis of grape samples by commercial laboratories to quantify smoke taint marker compounds (i.e., volatile phenols and their glycoconjugates), but this can be prohibitively expensive for some producers [6,7]. Alternatively, grapes can be harvested and vinified so that sensory analysis can be conducted in-house. However, depending on the timing of smoke exposure, these approaches may not inform decision-making within the time-constraints of vintage.

To date, there has been little research into the use of affordable in-field technology to assess grapevine smoke contamination. Recently, the authors’ group published a study evaluating short-range remote sensing in the thermal and near-infrared spectrum, combined with machine learning, as a novel approach to assessing smoke contamination in grapevine leaves, berries, and wines, with high levels of accuracy [5]. These tools may support rapid decision-making, enabling the implementation of management strategies that reduce the risk of contamination carrying over into wine, as smoke taint.

Electronic noses (e-nose) are comprised of an array of metal oxide semiconductor sensors (MOS) sensitive to different gases that can measure a variety of volatiles in the environment [8]. Early developments of e-noses involve arrays of 5–8 tin-oxide type of MOS sensors, requiring the use of sealed chambers and/or a complete setup of different devices to heat the sample and obtain headspace to be injected in the e-nose chamber, which has made the e-noses non-portable as they require a laboratory setup [9,10]. Some studies have explored different signal extraction methods, such as the Lorentzian model, which has resulted in a powerful and rapid-response technique [11]. Ayhan et al. [12] explored the fluctuation-enhanced sensing method to detect and classify gases with improved accuracy when developing classification models using machine learning algorithms. Some applications include medical diagnostics [13], space shuttles and stations [14,15,16], crime and security [17], and food and beverages, such as rapeseed to detect volatile compounds in pressed oil [18], wine [19], and beer [20], among others. The latter study describes a low-cost e-nose developed with nine gas sensors to assess the aroma profile of beers coupled with machine learning modeling. Examples of the implementation of e-noses for food science can be found from early literature reviews [21] through the implementation of disease diagnostics [22], more recent applications to assess food quality [23], meat quality assessment [24], for food control [23], assessment of food authentication and adulteration [25], and for the wine industry [26,27,28,29,30]. However, the e-noses used in the past range in complexity, accessibility to users, and cost.

Low-cost e-noses can be used in the field to assess smoke contamination levels coupled with the internet of things (IoT) for data transmission and analysis from different locations or nodes within vineyards. However, a more efficient approach could be to mount e-noses to assess gases in different parts of vineyards and to generate geo-referenced maps of these gases on unmanned terrestrial vehicles (UTV), robots [31], or unmanned aerial vehicles (UAV) [32]. The levels of smoke-related contaminants could be modeled using machine learning algorithms to infer the levels of contaminants in berries, and therefore, the risk of smoke taint in the final wine. However, they could not be used to directly “sniff” these contaminants from bunches since smoke-derived volatile compounds are rapidly metabolized in berries, leading to the formation of glycoconjugates, which are odorless [2,5,6,7,33,34,35].

**Figure 1:**
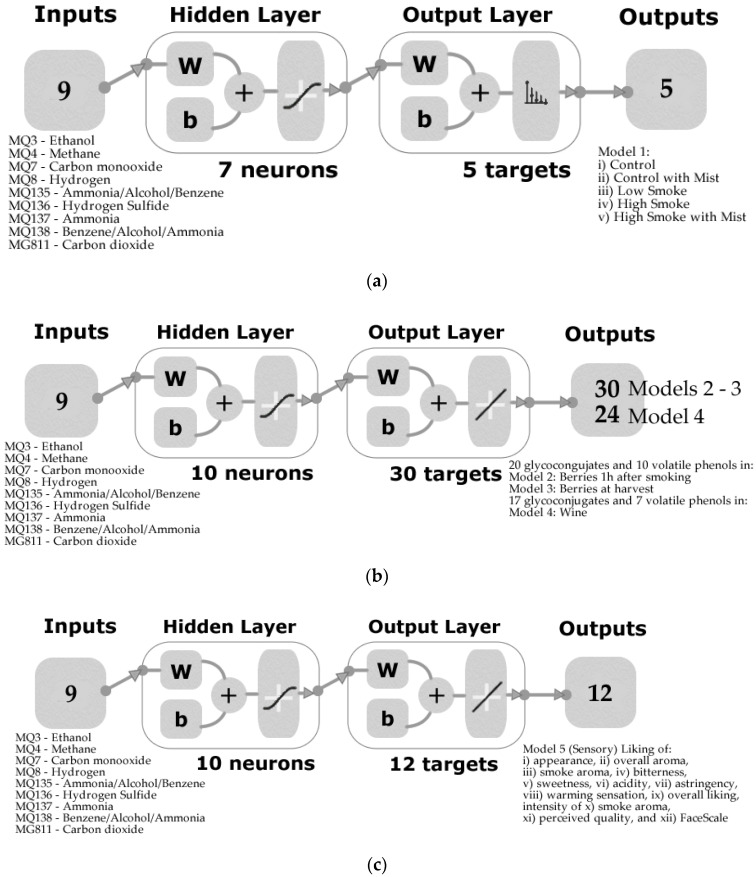
Model diagrams of the two-layer feedforward networks for (**a**) Model 1 for pattern recognition to classify samples into the five treatments using seven neurons, (**b**) Models 2–4 for regression to predict 20 glycoconjugates and 10 volatile phenols (Table 2) in Model 2: berries 1 h after smoke, Model 3: berries at harvest, and Model 4: wine, and (**c**) Model 5 for regression to predict 12 different sensory responses using 10 neurons. Abbreviations: W: weights, b: bias.

This study evaluated the potential for low-cost e-noses to be used to assess wines made from grapes exposed to different levels (densities) of smoke. The e-nose measurements were used as inputs in machine learning modeling strategies, and the concentrations of smoke taint marker compounds in berries and wines used as targets. Further, targets were obtained from a sensory analysis trial, during which consumers assessed the wines made from each treatment. In total, five machine learning models were created based on e-nose data to assess (i) the level of contamination in grapevines related to smoke exposure from wine samples using classification models (Model 1); (ii) to evaluate smoke-related compounds from wines, such as 20 glycoconjugates and 10 volatile phenols in berries after 1 h smoke (Model 2), (iii) smoke-related compounds in berries measured at harvest (Model 3), (iv) for wines made from treatments (Model 4), and (v) consumer sensory analysis using 12 wine descriptors (Model 5; Figure 1). The models obtained were of high accuracy, which could allow the implementation of this artificial intelligence (AI) technology in the winemaking process to assess the effect of ameliorating management techniques in the field (Model 1) through micro-vinifications, to assess the best timing for skin contact during fermentation for red wines, the addition of activated carbon to adsorb smoke-related compounds, wine filtration using membranes, reverse osmosis, and other commercial fining agents, among others [34,35].

Not only could the implementation of this technique help winemakers evaluate the different amelioration techniques mentioned above, but it could also monitor almost real-time changes in the aroma profiles of wine and assess which technology could best maintain a certain quality or style target.

This paper described how the e-nose was implemented for the different treatments and wine samples used and the specific machine learning algorithms used to develop five machine learning models with their respective analyses for accuracy and performance. A discussion on potential applications of the e-nose and models was also described for the wine industry to monitor and reduce smoke taint in wines.

## 2. Materials and Methods

### 2.1. Description of Treatments and Wine Samples

Field trials involving the application of smoke and/or in-canopy misting to Cabernet Sauvignon grapevines have been reported previously [3]. Briefly, three different smoke treatments were applied to vines (at approximately 7 days post-véraison): (i) low-density smoke exposure (LS), (ii) high-density smoke exposure (HS), and (iii) high-density smoke exposure, with in-canopy misting (HSM). Two controls were also included: (iv) a control without misting (C; no smoke treatment) and (v) a control with misting (CM; no smoke treatment). Treatments were applied to six adjacent vines, except for HSM, which was applied to five adjacent vines (i.e., one vine was missing). Smoke treatments involved exposure of grapevines to straw-derived smoke using a purpose-built tent for 1 h. At least one buffer vine separated treatments. The wine was subsequently produced on a small scale (i.e., ~5 kg per fermentation, performed in triplicate for each treatment), as described previously [3].

### 2.2. Electronic Nose

Wine samples were measured (in triplicate) using a portable, user-friendly, and low-cost e-nose, comprising nine different sensors, which were sensitive to different gases, as mentioned in Table 1, plus a humidity and temperature sensor (AM2320; Guangzhou Aosong Electronics Co., Ltd., Guangzhou, China). Sensor details have already been reported [20]. A total of 100 mL of wine was poured into a 500 mL beaker, and the e-nose was placed on top of the container for 1 min to capture the gases present in the sample. The e-nose was calibrated for 20–30 s before and after measuring each sample to reset the readings to baseline. Values from all sensors were automatically recorded in a comma-separated values (.csv) file to facilitate analysis.

### 2.3. Chemical Analysis of Glycoconjugates and Volatile Phenols

Volatile phenols (Table 2) were evaluated in wine samples using stable isotope dilution analysis (SIDA) methods, as previously described [15,17,18,19]. Isotopically labeled standards of d_3_-guaiacol, d_3_-4-methylguaiacol, d_7_-*o*-cresol, and d_3_-syringol were prepared in house by the Australian Wine Research Institute’s (AWRI) Commercial Services Laboratory (Adelaide, Australia) using published methods [15,17,18]. Measurements were performed using an Agilent 6890 gas chromatography coupled to a 5973 mass-spectrometer (Agilent Technologies, Forest Hill, VIC, Australia). The limit of quantitation for volatile phenols was 1–2 µg L^−1^.

A range of volatile phenol glycoconjugates (Table 2) was measured using high-performance liquid chromatography-tandem mass spectrometry (HPLC-MS/MS) according to stable isotope dilution analysis (SIDA) methods previously described [18,20]. The analysis was performed using an Agilent 1200 high-performance liquid chromatography (HPLC) equipped with a 1290 binary pump, coupled to an AB SCIEX Triple QuadTM 4500 tandem mass spectrometer, with a Turbo VTM ion source (Framingham, MA, USA). The preparation of the isotopically labeled internal standard d_3_-syringol gentiobioside has been previously reported [18,20]. The limit of quantitation for volatile phenol glycosides was 1 µg kg^−1^.

### 2.4. Sensory Evaluation-Consumer Test

A consumer test was conducted with participants (N = 31; age range: 21–59 years; 77% female and 23% male) constituted of staff and students from The University of Melbourne (UoM; Ethics ID: 1545786.2) that had been recruited via e-mail. According to the power analysis conducted using the SAS^®^ Power and Sample Size v. 14.1 software (SAS Institute Inc., Cary, NC, USA), the number of participants was enough to find significant differences between samples (power: 1 − β > 0.99). The session was carried out in the sensory laboratory of the Faculty of Veterinary and Agricultural Sciences (FVAS) in individual booths with uniform white light-emitting diode (LED) lights. Each booth was equipped with a tablet PC in which the Bio-Sensory Application (The University of Melbourne, Parkville, VIC, Australia) was set up with the questionnaire to gather consumer responses. The appearance, overall aroma, smoke aroma, bitterness, sweetness, acidity, astringency, a warming sensation, and overall liking were assessed on a likeness scale (i.e., dislike extremely—neither like nor dislike—like extremely). The levels of smoke aroma and perceived quality were rated on an intensity scale (i.e., absent-intense). Both liking and intensity measures were presented on a 15 cm non-structured continuous scale. In addition, emotional responses were recorded, using a 0–100 FaceScale, where 0 = sad 🙁, 50 = neutral 😐, and 100 = happy 😊. Samples were randomly assigned a 3-digit code, and 10 mL samples were served at room temperature (20 °C) in International Standard Wine Tasting Glasses (Bormioli Luigi, Fidenza, Italy). Samples were served in random order to avoid bias. Plain water and water crackers were used as palate cleansers between samples.

### 2.5. Statistical Analysis and Machine Learning Modeling

Analysis of variance (ANOVA) was conducted on e-nose data using XLSTAT (ver. 19.3.2, Addinsoft Inc., New York, NY, USA), and Tukey’s honest significant difference test (HSD; α = 0.05) was used to assess significant differences between treatments.

Machine learning modeling was performed based on artificial neural networks (ANN) for both pattern recognition and regression models, using codes written in Matlab^®^ R2019b (Mathworks, Inc., Natick, MA, USA) developed to test 17 different training algorithms. Five distinct models were developed using 20 data points from the peak of the e-nose outputs (nine sensors) as inputs. Model 1 (pattern recognition) used the scaled conjugate gradient training algorithm to classify the wine samples into the five different treatments: (i) LS, (ii) HS, (iii) HSM, (iv) C, and (v) CM. All four regression models were developed using the Levenberg Marquardt algorithm. Model 2 consisted of the use of the 20 glycoconjugates and 10 volatile phenols (Table 2) found in berries one hour after being exposed to smoke as targets. In comparison, Model 3 used the same 20 glycoconjugates and 10 volatile phenols in berries but measured at harvest. The targets used for Model 4 were 17 glycoconjugates and seven volatile phenols analyzed in the wine samples (Table 2). On the other hand, Model 5 was developed to predict 12 sensory responses, using the liking of (i) appearance, (ii) overall aroma, (iii) smoke aroma, (iv) bitterness, (v) sweetness, (vi) acidity, (vii) astringency, (viii) a warming sensation, (ix) overall liking, and (x) the intensity of (i) smoke aroma, (ii) perceived quality, and (iii) the FaceScale emotional response as targets.

All inputs and targets were normalized from −1 to 1. Data were divided randomly for all ANN models, with 60% of the data being used for the training stage, 20% for validation, and 20% for testing. Model 1 used a cross-entropy loss to test performance, while Models 2–5 were based on means squared error (MSE). Figure 1 shows the diagrams for Model A (Figure 1a), Models 2–4 (Figure 1b), and Model 5 (Figure 1c); all models consisted of a two-layer feedforward network with the hidden layer using a tan-sigmoid function and the output layer using softmax neurons (Model 1) and a linear transfer function (Models 2–5). A trimming test (data not shown) was performed to find the optimal number of neurons (3, 5, 7, 10) to get the best performance. Statistical data reported for regression models to assess under- or overfitting consist of the correlation coefficient (R), slope (b), MSE, and determination coefficient (R^2^); the latter was calculated using the curve fitting tool found in Matlab^®^.

## 3. Results

### 3.1. Electronic Nose Results

**Figure 2:**
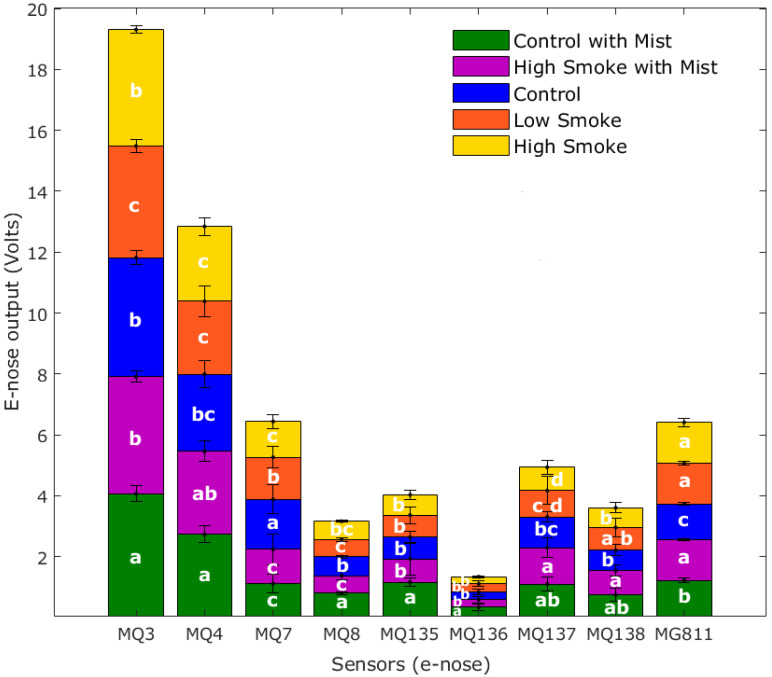
Mean values of the electronic nose outputs showing the letters of significance from the ANOVA and Tukey *post hoc* test (α = 0.05). Sensors: MQ3 = ethanol, MQ4 = methane, MQ7 = carbon monoxide, MQ8 = hydrogen, MQ135 = ammonia/alcohol/benzene, MQ136 = hydrogen sulfide, MQ137 = ammonia, MQ138 = benzene/alcohol/ammonia, MG811 = carbon dioxide.

Figure 2 shows the results from the ANOVA for the e-nose responses. It can be observed that there were significant differences (*p* < 0.05) between samples in the outputs from all nine sensors that integrated the e-nose. Ethanol gas (MQ3) presented the highest values for all wine samples with CM (mean = 4.07 V) being significantly different from HSM (mean = 3.85 V), HS (mean = 3.82 V), and C (mean = 3.92 V), and these from LS (mean = 3.66 V). Hydrogen sulfide (MQ136) was the lowest for all samples, and CM (mean = 0.34 V) was significantly different from all other samples (means = 0.23–0.27 V). The CO_2_ sensor readings are inverse; therefore, higher Volts mean lower concentration; it can be observed that all the samples with smoke treatments (LS, HS, and HSM) had the lowest CO_2_ and presented significant differences with control samples (CM and C).

Table 3 shows the minimum, maximum, and average values of the glycoconjugates and volatile phenols detected in berries one hour after smoking, in berries at harvest, and wine. It can be observed that there was a wide range of values for all of the compounds, which indicated these were adequate samples to be used for machine learning modeling and to detect smoke contamination.

Table 4 shows the minimum, maximum, and average values of the responses from the sensory session conducted with consumers when evaluating the wines. It can be observed that the results from all attributes were within the whole range of the scales used for liking and appearance (0–15) and FaceScale (0–100), which made the data suitable to be used for machine learning modeling.

### 3.2. Machine Learning Models

**Figure 3:**
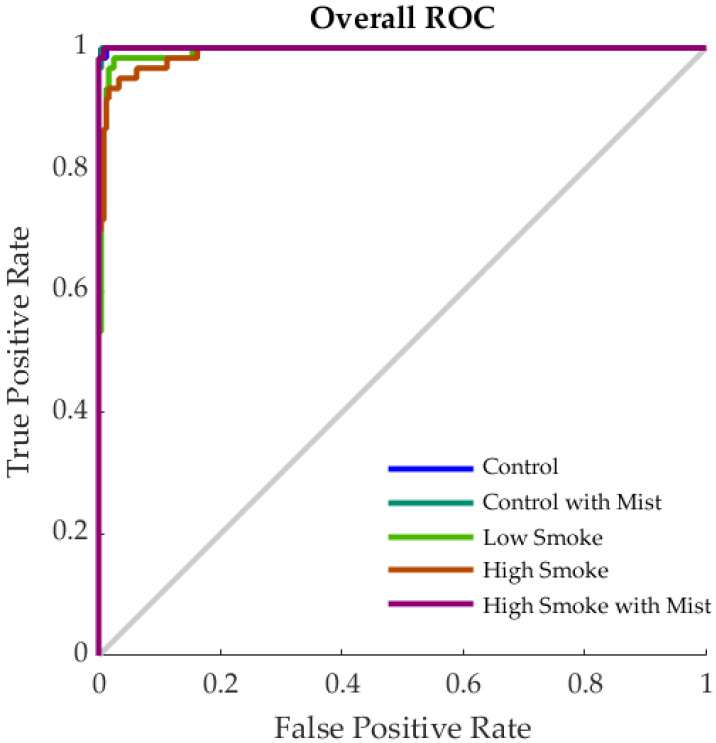
Receiver operating characteristic (ROC) curve for Model 1 to classify wine samples into the five different smoke treatments.

Table 5 shows the statistical results from Model 1 for the classification of the samples into the five different treatments. It can be observed that there was a high accuracy for all stages (>90%) and 97% for the overall model. According to the performance values, there were no signs of overfitting, as the training stage had a cross-entropy value lower than the validation and testing, and these two had similar performance. In Figure 3, the results from the receiver operating characteristic (ROC) curve are shown. This graph depicted the sensitivity (true positive rate) and specificity (false positive rate) of the overall model, with optimal operating points of 98%, 100%, 93%, 93%, and 98% for C, CM, LS, HS, and HSM, respectively.

**Figure 4:**
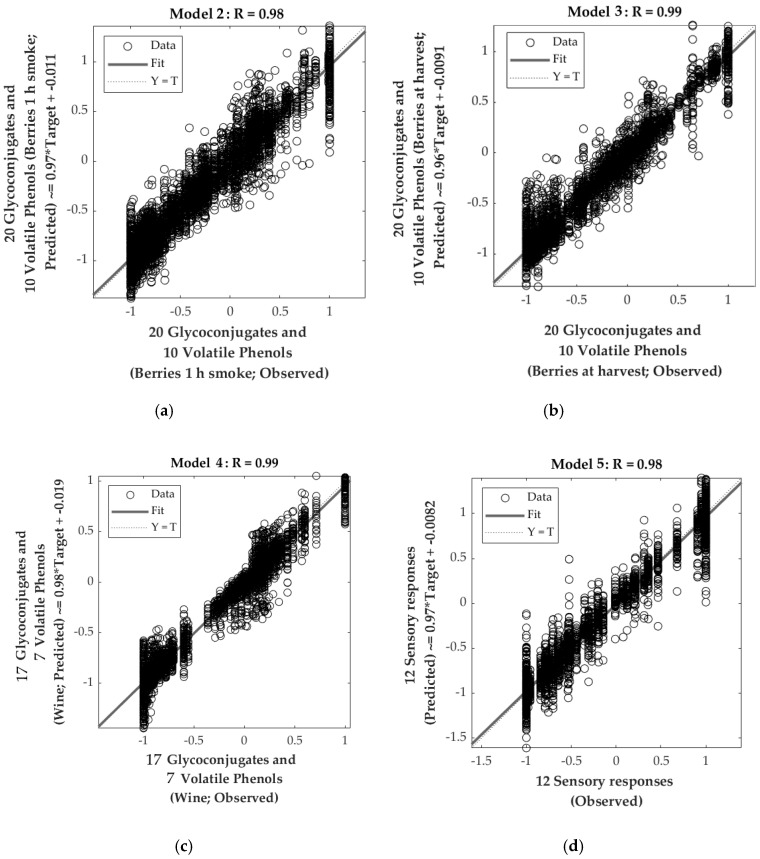
The overall correlation of the models to predict 20 glycoconjugates and 10 volatile phenols (Table 2) of (**a**) Model 2: berries after 1 h smoking, (**b**) Model 3: berries at harvest; (**c**) 17 glycoconjugates and seven volatile phenols of Model 4: wine. (**d**) Shows the Model 5 to predict 12 sensory descriptors obtained in a consumer test (Figure 1c).

Table 6 depicts the statistical data for the four regression models. Model 2 had very high overall correlation and determination coefficients (R = 0.98; Figure 4a; R^2^ = 0.95). The close value of the validation and training correlation coefficients (R = 0.96 and R = 0.98, respectively), along with the fact that the performance of the training stage (MSE = 0.01) was lower than that of the validation and testing (MSE = 0.03 and MSE = 0.02, respectively), showed that there were no signs of under- or overfitting. Models 3 and 4 had similar statistical values, both with high accuracy (Model 3: R = 0.99; Figure 4b; R^2^ = 0.97; Model 4: R = 0.99; Figure 4c; R^2^ = 0.98). These models also showed no signs of under- or overfitting. On the other hand, Model 5 also had a very high overall accuracy (R = 0.98; Figure 4d; R^2^ = 0.96) with similar performance values for validation and testing (MSE = 0.04) and higher than that of the training stage (MSE = 0.02). All models presented a slope close to the unity (b ~ 1) for all stages (Figure 4).

## 4. Discussion

Nowadays, the only alternative for grape growers is to apply potential amelioration techniques before the bushfires and hope for the best since there are limited tools that can be applied in the field or at the winemaking stage, which can render results in near real-time for proper decision-making [35,36]. Recently, non-invasive devices have been proposed using infrared thermal imaging to assess contaminated grapevine canopies in the field and smoke taint in berries and wines using near-infrared spectroscopy [5,33]. The research presented in this paper has contributed to the potential implementation of new and emerging sensor technologies and modeling strategies using machine learning in the viticulture and winemaking industry. These low-cost e-noses could become a game-changer for the management of smoke contamination and taint in berries and wines due to bushfires.

In general, previous applications of e-noses in the wine industry have been implemented mainly for the analysis of grapes and crushing methods [37], improvement of maceration and fermentation processes [38], to monitor the aging of wine in barrels [39,40,41], geographical classification [42], wine spoilage [28,43,44], and to assess correlations with human perception through sensory evaluation [27,29,45]. However, most of these studies have been based on multivariate data analysis and correlation analysis.

Low-cost sensors presented in this research, developed by integrating an array of gas sensors [20], could be used in the winery to assess the level of grapevine smoke exposure. In the present study, models were developed to evaluate the effects of different amelioration techniques (Model 1) for berries immediately after the bushfire event (Model 2), at harvest time (Model 3), and in the actual wines (Model 4). Since smoke-derived glycoconjugates in berries are difficult to detect using e-noses due to the binding of these compounds with sugars in the berries, these assessments need to be performed after the winemaking process, in which the compounds are released through the maceration and fermentation processes.

A further model (Model 5) developed to assess sensory characteristics of wines rapidly and objectively, which can be implemented in parallel with successful amelioration techniques to reduce smoke taint, such as the addition of activated carbon to wines or fining agents [2,34]. For the latter case, Model 5 will offer a near real-time assessment of the techniques used.

The advantages of implementing these models coupled with low-cost sensor technology are that grape growers and winemakers will not depend on random sampling, which may not render representative results, or external laboratory services, which may not deliver results in a timely manner due to being overwhelmed by large sample volumes that are delivered when concurrent bushfires occur. Knowing the levels of smoke-derived compounds and the effects on consumer appreciation in the winemaking process offer the following advantages: (i) rapid and user-friendly smoke taint determination; (ii) potential implementation of techniques to reduce smoke taint using activated carbon or fining agents on samples and re-test using the e-nose and models developed; (iii) sensory panel not required for assessments/modifications, minimizing the time for the commercial release of wines and economic impacts of smoke taint.

Further applications of these low-cost e-noses can be implemented to assess the maturity of grapes in the field, specifically through the alcohol-based sensors. The latest research has shown that ethanol is released from grape berries when they become oxygen stressed [46]. So, being able to assess when cell death begins would be a useful tool in monitoring berry health and fruit ripening potential. These processes of berry cell death assessment can be done non-destructively by near-infrared spectroscopy and machine learning modeling [47] or by tracking ethanol release from grapevine bunches through the implementation of low-cost e-noses in the field using sensor networks or as a payload of low altitude unmanned aerial vehicle (UAV) surveys [48,49].

## 5. Conclusions

Low-cost e-nose sensor technology coupled with machine learning offers the advantage of easy implementation in field conditions using sensor networks or in the winery. Machine learning models obtained could make available valuable information to winemakers and winegrowers for the decision-making process to produce commercial wines by minimizing smoke taint. An artificial intelligence system can be implemented based on sensor technology and machine learning developed here to obtain the least tainted wine or to target specific sensory aroma profiles to take advantage of the decontamination process to maximize the likability of wines.

## Figures and Tables

**Table 1:** Sensors, attached to the electronic nose, and the gasses they are sensitive to.

Sensor Name	Gases	Manufacturer
MQ3	Ethanol	Henan Hanwei Electronics Co., Ltd., Henan, China
MQ4	Methane	
MQ7	Carbon monoxide (CO)	
MQ8	Hydrogen	
MQ135	Ammonia, alcohol, and benzene	
MQ136	Hydrogen sulfide	
MQ137	Ammonia	
MQ138	Benzene, alcohol, and ammonia	
MG811	Carbon dioxide (CO_2_)	

**Table 2:** List of glycoconjugates and volatile phenols, their abbreviation, and the sample in which they were measured.

Compound	Abbreviation/Label	Sample
Glycoconjugates		
Syringol gentiobiosides	SyGG	Berries/Wine
Syringol glucosides	SyMG	Berries/Wine
Syringol pentosylglucosides	SyPG	Berries/Wine
Cresol glucosylpentosides	CrPG	Berries/Wine
Cresol gentiobioside	CrGG	Berries
Cresol glucosides	CrMG	Berries
Cresol rutinosides	CrRG	Berries/Wine
Guaiacol pentosylglucosides	GuPG	Berries/Wine
Guaiacol gentiobiosides	GuGG	Berries/Wine
Guaiacol rutinosides	GuRG	Berries/Wine
Guaiacol glucosides	GuMG	Berries/Wine
Methylguaiacol pentosylglucosides	MGuPG	Berries/Wine
Methylguaiacol rutinosides	MGuRG	Berries/Wine
Methylguaiacol glucosides	MGuMG	Berries
Methylsyringol gentiobiosides	MSyGG	Berries/Wine
Methylsyringol pentosylglucosides	MSyPG	Berries/Wine
Phenol rutinosides	PhRG	Berries/Wine
Phenol gentiobiosides	PhGG	Berries/Wine
Phenol pentosylglucosides	PhPG	Berries/Wine
Phenol glucosides	PhMG	Berries/Wine
**Volatile Phenols**		
Guaiacol	Guaiacol	Berries/Wine
4-Methylguaiacol	4-Methylguaiacol	Berries/Wine
Phenol	Phenol	Berries
*o*-Cresol	*o*-Cresol	Berries/Wine
Total *m*/*p*-cresols	Total *m*/*p*-cresol	Berries
*m*-Cresol	*m*-Cresol	Berries/Wine
*p*-Cresol	*p*-Cresol	Berries/Wine
Syringol	Syringol	Berries/Wine
4-Methylsyringol	4-Methylsyringol	Berries/Wine
Total cresols	Cresols	Berries

**Table 3:** Minimum (Min), maximum (Max), and mean values of the glycoconjugates (berries: µg kg^−1^; wine: µg L^−1^) and volatile phenols (µg L^−1^) detected in berries and wine.

Compound	Berries 1 h After Smoking			Berries at Harvest			Wine		
	Min	Max	Mean	Min	Max	Mean	Min	Max	Mean
Syringol gentiobioside	2.37	56.93	15.42	6.30	772.81	186.55	10.43	582.11	152.58
Syringol monoglucoside	0.14	26.97	6.38	2.65	68.34	19.22	0.36	14.54	4.26
Syringol pentosylglucosides	0.76	4.52	1.79	6.41	369.14	88.76	1.70	103.37	27.73
Cresol glucosylpentosides	8.07	47.12	18.13	41.69	1395.52	382.63	0.40	17.67	5.28
Cresol gentiobioside	0.18	0.71	0.45	1.94	6.46	3.55	NA	NA	NA
Cresol monoglucoside	0.24	61.87	16.36	0	35.47	8.70	NA	NA	NA
Cresol rutinoside	1.62	13.34	4.90	3.11	122.07	38.35	2.91	133.85	40.55
Guaiacol pentosylglucosides	2.29	25.61	7.57	15.76	1233.46	268.39	5.30	330.36	80.47
Guaiacol gentiobioside	0.05	1.38	0.40	0.54	67.44	16.33	0.30	2.81	0.99
Guaiacol rutinoside	0	1.35	0.48	1.13	32.03	9.97	0	48.60	15.24
Guaiacol monoglucoside	0.03	30.04	7.07	1.22	30.25	7.15	0.12	12.60	3.46
Methylguaiacol pentosylglucosides	0.55	11.51	3.29	6.79	266.50	57.32	1.43	51.79	12.72
Methylguaiacol rutinoside	0.60	5.58	1.89	6.45	153.06	44.36	0.79	40.92	11.97
Methylguaiacol monoglucoside	0	0	0	0.94	11.52	3.89	NA	NA	NA
Methylsyringol gentiobioside	0.33	13.34	3.49	2.53	302.51	72.52	0.15	30.69	7.41
Methylsyringol pentosylglucosides	0.07	0.39	0.17	1.57	34.84	10.36	0.20	8.35	2.46
Phenol rutinoside	0.31	3.78	1.26	3.75	175.57	53.28	1.42	77.58	23.40
Phenol gentiobioside	0.01	0.61	0.15	0	28.54	6.57	0.08	6.22	1.70
Phenol pentosylglucosides	1.44	24.97	7.02	16.21	812.10	215.13	0.53	22.59	6.31
Phenol monoglucoside	0.04	2.55	0.63	0.99	21.52	5.65	0.74	43.48	11.86
Guaiacol	2.39	139.72	41.57	2.06	12.97	5.08	0	39.00	11.73
4-Methylguaiacol	3.54	27.72	9.50	3.52	4.45	3.80	0	5.00	1.40
Phenol	1.40	85.68	21.12	1.26	26.38	9.61	NA	NA	NA
*o*-Cresol	1.65	54.02	16.31	1.74	8.08	4.02	0	14.00	4.87
Total *m*/*p*-cresol	0.56	63.07	16.01	0.52	7.71	2.99	NA	NA	NA
*m*-Cresol	1.90	45.07	12.08	1.84	5.89	3.24	0	14.00	4.53
*p*-Cresol	0	18.00	4.38	0	2.04	0.44	0	9.00	2.60
Syringol	5.17	180.31	47.67	9.32	13.77	11.73	1.00	6.00	3.13
4-Methylsyringol	1.83	24.36	6.62	1.75	2.11	1.83	0	0	0
Total cresols	2.22	117.08	32.32	2.26	15.79	7.01	NA	NA	NA

Abbreviations: NA: Not applicable. Values <1 (µg L^−1^ and µg kg^−1^) are considered as below the limit of detection. However, actual values were included in the modeling strategies.

**Table 4:** Minimum (Min), maximum (Max), and mean values of the sensory session responses for wine tasting.

Data/Sensory Attribute	Min	Max	Mean
Appearance liking	0.45	15.00	7.19
Overall aroma liking	0.30	14.85	6.21
Smoke aroma intensity	0	15.00	4.98
Smoke aroma liking	0	15.00	4.72
Bitter liking	0.30	15.00	5.98
Sweet liking	0	14.70	6.16
Acidity liking	0	14.70	6.23
Astringency liking	0.30	15.00	6.27
Warming liking	0.30	15.00	6.20
Overall liking	0.30	14.85	6.07
Perceived quality	0	14.85	5.66
FaceScale	0	99.00	42.15

**Table 5:** Statistical results from the pattern recognition model (Model 1) to classify samples into five different treatments (control, control with mist, low smoke, high smoke, and high smoke with mist).

Stage Model 1	Samples	Accuracy	Error	Performance (Cross-Entropy)
Training	180	99%	1%	0.01
Validation	60	93%	7%	0.04
Testing	60	92%	8%	0.05
Overall	300	97%	3%	-

**Table 6:** Statistical results from the four regression models (Models 2–4: glycoconjugates and volatile phenols; Model 5: sensory) showing the correlation coefficient (R), determination coefficient (R^2^), slope (b), and performance based on means squared error (MSE) for each stage.

Stage/ Model 2 (Berries 1 h Smoke)	Samples	Observations	R	R^2^	b	Performance (MSE)
Training	180	5400	0.98	0.96	0.96	0.01
Validation	60	1800	0.96	0.92	0.97	0.03
Testing	60	1800	0.97	0.95	0.97	0.02
Overall	300	9000	0.98	0.95	0.97	-
**Stage/** **Model 3** **(Berries at Harvest)**	**Samples**	**Observations**	**R**	**R^2^**	**b**	**Performance** **(MSE)**
Training	180	5400	0.99	0.98	0.97	0.01
Validation	60	1800	0.98	0.95	0.96	0.02
Testing	60	1800	0.98	0.97	0.95	0.01
Overall	300	9000	0.99	0.97	0.96	-
**Stage/** **Model 4** **(Wine)**	**Samples**	**Observations**	**R**	**R^2^**	**b**	**Performance** **(MSE)**
Training	180	4320	0.99	0.99	0.99	<0.01
Validation	60	1440	0.98	0.95	0.96	0.02
Testing	60	1440	0.98	0.96	0.95	0.01
Overall	300	7200	0.99	0.98	0.98	-
**Stage/** **Model 5** **(Wine Sensory)**	**Samples**	**Observations**	**R**	**R^2^**	**b**	**Performance** **(MSE)**
Training	180	2160	0.98	0.97	0.97	0.02
Validation	60	720	0.97	0.94	0.97	0.04
Testing	60	720	0.97	0.94	0.97	0.04
Overall	300	3600	0.98	0.96	0.97	-
